# Mortality- and Health-Related Factors in a Community-Dwelling of Oldest-Older Adults at the Age of 90: A 10-Year Follow-Up Study

**DOI:** 10.3390/ijerph17249584

**Published:** 2020-12-21

**Authors:** Yoshiaki Nomura, Mieko Shimada, Erika Kakuta, Ayako Okada, Ryoko Otsuka, Yasuko Tomizawa, Chieko Taguchi, Kazumune Arikawa, Hideki Daikoku, Tamotsu Sato, Nobuhiro Hanada

**Affiliations:** 1 Department of Translational Research, School of Dental Medicine, Tsurumi University, Yokohama 230-8501, Japan; okada-a@tsurumi-u.ac.jp (A.O.); otsuka-ryoko@tsurumi-u.ac.jp (R.O.); hanada-n@tsurumi-u.ac.jp (N.H.); 2 Department of Dental Hygiene, Chiba Prefectural University of Health Sciences, Chiba 261-0014, Japan; mieko.shimada@cpuhs.ac.jp; 3 Department of Oral Bacteriology, School of Dental Medicine, Tsurumi University, Yokohama 230-8501, Japan; kakuta-erika@tsurumi-u.ac.jp; 4 Department of Cardiovascular Surgery, Tokyo Women’s Medical University, Tokyo 162-8666, Japan; tomizawa.yasuko@twmu.ac.jp; 5 Department of Preventive and Public Oral Health, School of Dentistry at Matsudo, Nihon University, Matsudo 470-2101, Japan; taguchi.chieko@nihon-u.ac.jp (C.T.); arikawa.kazumune@nihon-u.ac.jp (K.A.); 6 Iwate Dental Association, Morioka 020-0045, Japan; dai-koku@nifty.com (H.D.); tamosato-dent@k-2inc.jp (T.S.)

**Keywords:** mortality, self-assessed chewing ability, serum albumin, ADL, physical performance

## Abstract

Mortality is obviously intended for epidemiological studies of community-dwelling older adults. There are several health-related factors associated with nutritional status and mortality. The aim of this study was to elucidate the risk factor for mortality in community-dwelling oldest-older adults at the age of 90 and clarify the structure of health-related factors associated with mortality. A 10-year follow-up study was performed for 93 subjects at the age of 90. The mean and median of their survival days were 2373 and 2581 days for women, and 1694 and 1793 days for men. By Cox’s proportional hazards model, health-related factors associated with mortality were self-assessed for chewing ability, activities of daily living (ADLs), serum albumin, total cholesterol, serum creatinine, and gripping power for women but not for men. These factors interacted with each other, and the association of these factors was different in women and men. Self-assessed chewing ability was a powerful risk factor for mortality in women at the age of 90. It acted independently from nutritional status. For older adults, addressing healthy food choices together with improved oral functions is useful. However, risk factors for mortality may depend on the life stage of subjects. To investigate the risk factor for the mortality, the life course approach is necessary.

## 1. Introduction

Mortality is obviously intended for epidemiological studies of community-dwelling older adults. Many health-related factors associated with mortality interact with each other [1,2,3,4,5,6]. Therefore, health-related factors associated with mortality comprise a complex structure.

Sufficient nutritional status is essential for maintaining health in older adults. A positive ageing process depends on adequate nutritional status [7]. Poor nutritional status leads to adverse health outcomes [8,9]. It results in functional decline and frailty [10,11] and has been suggested to be a risk for mortality [8,9].

Oral health status has a recondite impact on nutritional status. Food consistency and food choice have been adapted to oral status [12]. The association between impaired masticatory function and deficient dietary intake has been suggested [13,14]. Suboptimal nutritional status after impaired oral function can result in chronic diseases [15]. In addition, impaired oral function and dysphagia increases the risk of aspiration pneumonia and choking [16,17]. Several studies have suggested that self-assessed chewing ability or mastication deficiency is a risk for mortality [18,19,20,21]. However, for addressing oral health and nutrition, well-designed studies are still not enough [22].

Nutritional status and diet quality effect the physical performance and activities of daily living (ADLs) [9,23,24], as well as oral health-related to physical performance and ADLs [25,26,27]. Therefore, health-related factors can interact with each other and impact mortality.

Regarding community-dwelling older adults, evidence has been accumulated for the numerous risk factors associated with mortality. Studies have found that specific health-related factors are regarded as risks after adjustment for common risk factors. An investigation was performed within specific health-related factors, including nutrition [28,29], oral health [30,31,32,33,34,35,36,37,38], and physical performance [39,40,41,42,43,44,45]. In addition to this risk evaluation, a comprehensive assessment for the risk factors for mortality is necessary for planning health promotion for older adults [46,47], decision of priority of intervention [48,49,50], and health-related political decision-making.

In 1997, the Japanese Ministry of Labor and Health directed and supported a survey of 80-year-old people residing in four areas of Japan. The aims of the survey were to investigate the relationship between oral health and systemic health in 80-year-old adults. Iwate Prefecture, located in the northern region of Japan, was one of the areas participating in this survey. The survey items were common to the baseline study. Then, four areas were independently carried out in the follow-up study. Iwate Prefecture conducted the 5-year follow-up (85 years of age), 10-year follow-up (90 years of age), and 20-year follow-up (100 years old). Previous reports had shown the importance of chewing ability for mortality [51,52]. In this study, all the data in the 10-year follow-up study (90 years of age) were analyzed, including chewing ability.

We assessed the association between mortality and health status via blood tests, including reflection of nutritional status, oral health status via self-assessed chewing ability, physical performance, and activities of daily living (ADLs). The magnitude of health-related factors pertinent to mortality were investigated. In addition, the correlation between mortality and these health-related factors were summarized.

The aim of this study was to elucidate the risk factor for mortality in community-dwelling oldest-older adults at the age of 90 and clarify the structure of health-related factors associated with mortality.

## 2. Materials and Methods

### 2.1. Setting

A 20-year follow-up survey was conducted on 80-year-old subjects living in 10 districts managed by one health center in Iwate Prefecture in northern Japan. For 20 years, heath examinations were conducted three times in 1997 for the 80-year-old subjects, in 2002 at the age of 85, and 2007 at the age of 90. Oral examination, physical performance measures, blood tests and questionnaire surveys were conducted as health examinations. Other than these three-times survey, no health examinations were conducted. In this study, data of subjects at the age of 90 were analyzed in a 10-year follow-up survey (2007–2017).

### 2.2. Study Population and Follow-Ups

In Japan, the Family Register Act obligated all Japanese citizens to register their birth, death, still birth, marriage, and divorce to municipality offices as residential registration.

In 2007, a complete count survey using cluster sampling based on residential registration in 10 districts was performed for all subjects over the age of 90 (i.e., born in 1917). Public health nurses visited the homes of 90-year-old individuals in order to recommend who should participate in the survey. A total of 100 subjects completed the survey. In October 2017, survival and dates of death were surveyed by the census register of 10 district [51,52,53].

### 2.3. Oral Examination

Oral examinations were carried out by a dentist. Dentists counted the number of remaining teeth and their locations and denture use was recorded.

### 2.4. Physical Performance Measures

To test normal walking speed, participants were asked to walk once on a straight 11 m walkaway on a flat floor at a normal speed. Walking speed was measured at a distance of 5 m between markers located 3 and 8 m from the start of the walkaway [54].

A Smedley type hand dynamometer (Yagami Co, Tokyo, Japan) was used to measure grip strength of both hands. A higher value of grip strength was employed [55].

One-leg standing time was an indicator of static balance. In the one-legged standing time test, we asked participants to stand on their preferred leg with their eyes open and hands down alongside their trunk and to look straight ahead at a dot 1 m in front of them. The duration of standing time was measured for up to 60 s, and the higher value from 2 trials was used in the analysis [54,55,56]. The stepping rate at sitting position was measured with a stepping rate counter. Participants were instructed to step on each leg as fast as possible for 10 s. The number of steps of both feet was totaled as the score [55].

### 2.5. Blood Tests and Blood Pressure

The health status of the subjects was evaluated using blood tests. Blood samples were collected before physical performance tests and oral examinations. Samples were kept on ice until the transport to a medical examination company. The inspected items were aspartate transaminase (AST), alanine aminotransferase (ALT), γ-glutamic pyruvic transaminase (γ-GTP), total protein, albumin, total cholesterol, triglyceride, high-density lipoprotein (HDL), low-density lipoprotein (LDL), creatinine, calcium, phosphate, glucose, hemoglobin A1c (HbA1c), IgG, IgA, and IgM. Blood pressure was measured by physician. Systolic pressure and diastolic pressure were recorded.

### 2.6. Questionnaire

#### 2.6.1. Self-Assessed Chewing Ability

Self-assessed chewing ability was investigated using the simple dichotomous choice: “Can you chew the following 15 foods?” The response solicited either a yes or no answer [20,52,53].

#### 2.6.2. Activities of Daily Living (ADLs)

Instrumental activity of daily living was evaluated using the Tokyo Metropolitan Institute of Gerontology (TMIG) index of competence [57]. Three subscales of the TMIG index were self-maintenance, intellectual activity, and social role. These subscales consisted of 5, 4, and 4 items. If subjects answered yes, it was counted as one point. A low point for self-maintenance (≤4 points), for intellectual activity (≤2 points), and for social role (≤2 points) were regarded as functional decline [58,59].

### 2.7. Statistical Analysis

#### 2.7.1. Item Response Theory (IRT)

A three-parameter logistic model was applied for the summary score of self-assessed chewing ability. Item response curves and item information curves were presented for 15 types of food [60,61,62,63,64]. R ver3.50 with the LTR and irtoys packages was used for IRT analysis.

#### 2.7.2. Survival Analysis

To calculate the hazard ratios, the Cox proportional hazards model was used. Survival rates were calculated using the Kaplan–Meier analysis. Differences in survival curves were evaluated using the log-rank test, the Breslow test, and the Tarone–Ware test [52,53]. These analyses were carried out using SPSS Statistics ver24.0 (IBM, Tokyo, Japan)

#### 2.7.3. Structural Equation Modeling (SEM) and Path Analysis

Structural equation modeling (SEM) was performed for the subscales of the TMIG index [52]. For factor-associated mortality, path analysis was performed [51]. The root mean square error of approximation (RMSEA) was used for the goodness of fit index [65,66]. SEM and path analysis were carried out using AMOS ver24.0 (IBM, Tokyo, Japan).

### 2.8. Ethics Approval

For the baseline survey, informed written consent was obtained from participants. This study was approved by the Ethics Committee of Tsurumi University School of Dental Medicine (Approval Number: 1515).

## 3. Results

### 3.1. Characteristics of the Subjects

The subjects that participated in this study were all born in 1917 and were 90 or 89 years old when the survey was conducted (September to October 2007). The health status evaluation of the subjects at the age of 90 was only one part of this survey. Among the 100 subjects who participated in this 2007 survey, 92 subjects could follow-up their health status after 10 years. The study population consisted of 58 men and 34 women. The mean and median of their survival days were 2373 and 2581 days for women, and 1694 and 1793 days for men. Their 95% confidential intervals (95% CI) were 2051 to 2696 days for the mean of women, 1857 to 3305 days for the median of women, 1365 to 2023 days for the mean of men, and 1079 to 2507 days for the median of men, respectively. Descriptive statistics of the all the variables analyzed in this study are shown Table S1.

### 3.2. Effect of Self-Assessed Chewing Ability for Mortality

#### 3.2.1. Analysis of Self-Assessed Chewing Ability Using IRT

**Figure 1:**
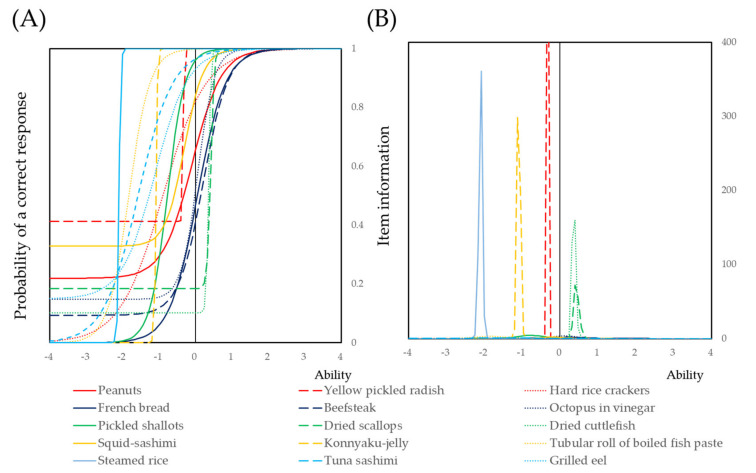
Item response curve **(A)** and item information curves **(B)** for 15 types of different food. Subjects participated in this study answered chewable “Yes” or “No” for different foods. The item response curves and item information curves were located in a backward direction to indicate easy to chew food. The curves with a forward direction indicate difficult to chew food.

Self-assessed chewing ability was analyzed via a three-parameter logistic model based on IRT. The item response curves and item information curves were shown in Figure 1. Among the 15 types of different food, steamed rice, Konnyaku jelly, yellow pickled radish, and dried cuttlefish had steep item response curves and high item information. The results of the three-parameter logistic model are shown in Table S2. The weighted sum of each item was calculated for the following analysis.

#### 3.2.2. The Effect of Self-Assessed Chewing Ability on Mortality

**Figure 2:**
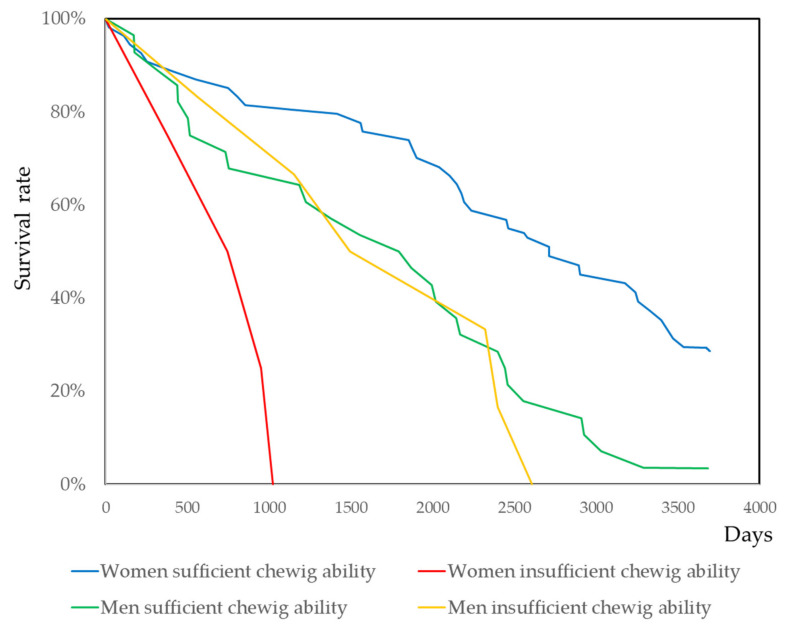
Survival curves by self-chewing ability. Sufficiency of chewing ability was determined by the above or less of the median value, the cut off was -1 of the ability shown in Figure 1. Statistical significance was evaluated using the log-rank test, the Breslow tests, and the Tarone–Ware test (*p* < 0.001, *p* = 0.001, and *p* < 0.001 for woman; *p* = 0.751. *p* = 0.828, and *p* = 0.992 for men, respectively).

The effect of self-assessed chewing ability on mortality was analyzed using Cox’s proportional hazard model, which was calculated via IRT. The results are shown in Table 1. Self-assessed chewing ability was statistically significant for women, but not significant for men. The number of remaining teeth and denture use were not significant. To describe the survival curves for the Kaplan–Meyer analysis, the ability of self-assessed chewing ability was dichotomized with the value’s median. The values were labelled as having sufficient or not sufficient chewing ability. The survival curves were illustrated in Figure 2. For women, the difference was statistically significant using the log-rank test, the Breslow test, and the Tarone–Ware test (*p* < 0.001, *p* = 0.001, and *p* < 0.001 for woman, respectively). However, it was not significant for men (*p* = 0.751. *p* = 0.828, and *p* = 0.992, respectively). Among the 15 types of food, statistical significance for the mortality of women was obtained for two foods: dried scallops and Konnyaku jelly. The survival curves for these two foods are shown in Figure S1. The results from Cox’s proportional hazard model analysis for the 15 foods are shown in Table S3.

### 3.3. Effect of Health Status by Blood Tests for Mortality

In this study, health status was evaluated via blood tests and blood pressure. For each blood test, Cox’s proportional hazard model was applied (Table 2). For women, albumin, total cholesterol, and creatine were statistically significant. However, there was no significant test for men. For self-assessed chewing ability and ADLs, the adjusted hazard ratio was calculated using these three blood tests. The results were shown in Table S4. After a serum albumin adjustment, self-assessed chewing ability was statistically significant for women. After adjusting for total cholesterol, ADL was significant for women.

### 3.4. Effect of ADLs for Mortality

#### 3.4.1. Structure of TMIG Index

**Figure 3:**
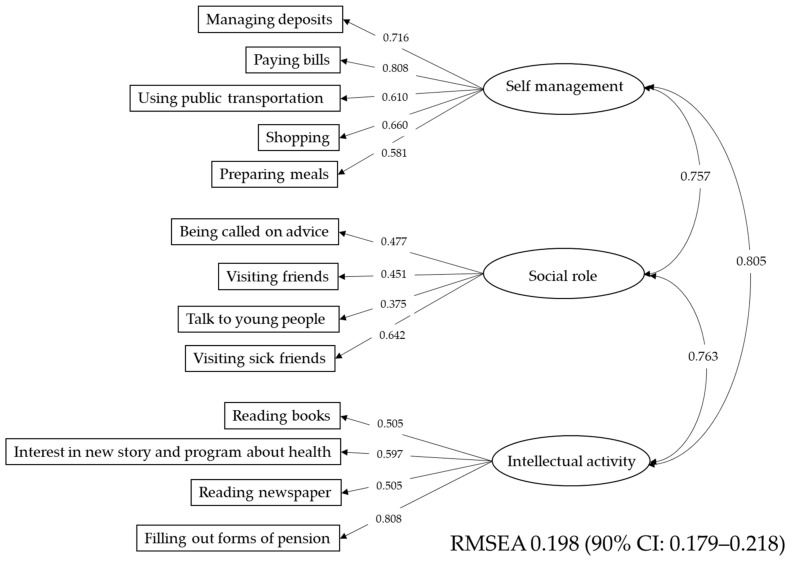
Structure of activities of daily living (ADLs). ADLs were evaluated using the TMIG index. The TMIG index consisted of 3 subscales: “self-management”, “social role”, and “intellectual activity”. These subscales were highly correlated with each other. TMIG index: Tokyo Metropolitan Institute of Gerontology Index, REMSEA: root mean square error of approximation.

The TMIG index consisted of three subscales. The structure of the TMIG index was analyzed using structural equation modeling (SEM). The result is shown in Figure 3. The subscales of “self-management”, “social role”, and “intellectual activity” were correlated with each other.

#### 3.4.2. Effect of ADLs on Mortality

**Figure 4:**
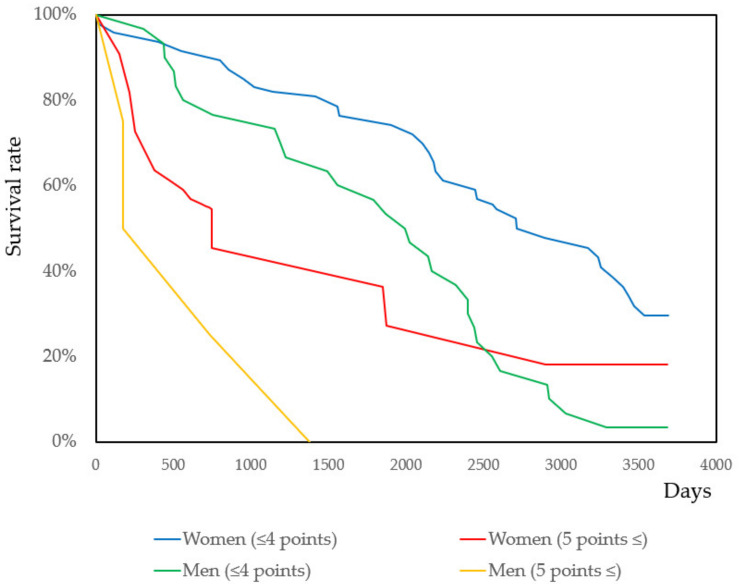
Survival curves by self-management as ADL. The subjects who participated in this study were classified as “self-management”, which was one of the subscales in the TMIG index. Statistical significance was evaluated using the log-rank test, the Breslow tests, and the Tarone–Ware test (*p* = 0.043, *p* = 0.008, and *p* < 0.014 for woman; *p* = 0.001. *p* = 0.001, and *p* = 0.001 for men, respectively).

Similarly, the effect of ADLs on mortality was analyzed using Cox’s proportional hazard model and the Kaplan–Meyer analysis. The results of Cox’s proportional hazard model are shown in Table 3. As there is no cutoff for the total TMIG index score, the three subscales have cutoffs. Subscales were treated as a dichotomous variable. In addition to total TMIG index scores, the “self-management” subscale was statistically significant for both women and men. Survival curves are illustrated in Figure 4. The survival curves of “intellectual activity” and “social role” are shown in Figure S1.

### 3.5. The Effect of Physical Fitness for Mortality

Hazed ratios for physical fitness were analyzed using Cox’s proportional hazard model. The results are shown in Table 4. Only female hand grip strength had statistical significance.

### 3.6. Overview of the Correlation between Health-Related Factors and Mortality

**Figure 5:**
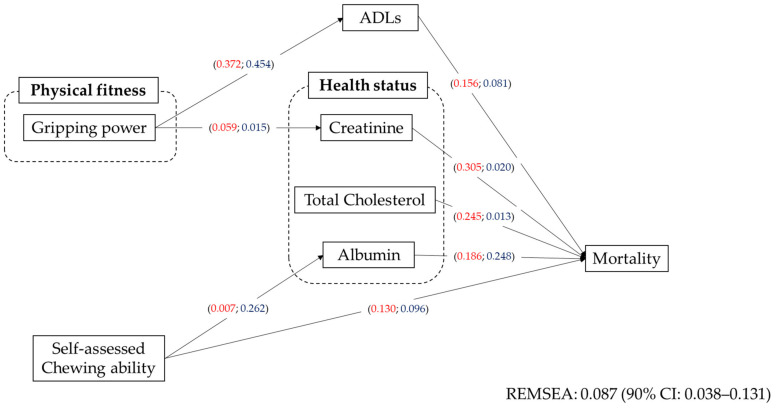
Overview of the effect of health-related factors on mortality for older adults at the age of 90. The correlation of health-related factors and mortality was analyzed using the path analysis. Numbers indicate the coefficients. Numbers in red are coefficients of women and in blue those of men. The fitness index was presented via REMSEA. REMSEA: root mean square error of approximation.

Based on the results described above, a path diagram was constructed (Figure 5). The red numbers indicate that the coefficient of women, whereas blue numbers indicate the coefficient of men. The coefficients were different between women and men. The effect of self-assessed chewing ability on serum albumin was higher in men and its direct effect on mortality was higher in women. Gripping power effected ADLs both in men and women. The effect of ADLs on mortality was higher in women. The effects of serum creatinine and total cholesterol were higher in women. In contrast, the effect of serum albumin on mortality was higher in men.

## 4. Discussion

In this study, we investigated health-related factors associated with the mortality of older adults at the age of 90. Self-assessed chewing ability, ADLs, serum albumin, total cholesterol, serum creatinine, and grip strength were significantly associated with mortality in women but not in men. These factors interacted with each other, and the association of these factors was different in men and women.

Several studies have shown that self-assessed chewing ability or self-assessed masticatory ability was associated with dental conditions [67,68,69]. However, the association was weak [68]. It is also associated with general health [69,70], social factors [70], and physical performance [71]. It is a stable indicator with minor variation over time [72]. It can be the predictor or a risk factor for mortality in older adults [18,19,20,21]. In this study, self-assessed chewing ability was statistically significant for women. In addition, adjusted hazard ratios of serum albumin, total cholesterol, and creatinine were also statistically significant in women but not in men. These results indicated that self-assessed chewing ability independently effected mortality, as evaluated via blood tests. Serum albumin reflected nutritional status [73] and is a well-known predictor for mortality [74,75]. When compared with men, association between self-assessed chewing ability and serum albumin was weak in women.

A decline in physical performance is a mortality risk [39,40,41,42,43,44,45]. Follow-up periods, age of subjects, and predictors varied between studies: short physical performance battery [39,40,41,42], walking speed (m/s), chair stand speed [43], comfortable walking speed, timed up and go, functional reach, and one-legged, single-legged, and double-legged [44] extensor strength and isokinetic leg extensor power [45]. In this study, some tests were a burden and dangerous for the older adults. Physical performance tests could not be completely performed. Among the various physical performance tests, a decline in hand grip strength was considered a mortality risk [76,77,78,79,80,81,82,83,84,85,86,87,88], especially for women. Further, it was associated with ADL (Figure 4). Hand grip strength tests had little risk and were easy to perform. It may help discover the subjects with deteriorating health [89].

ADL impairment was a risk for the mortality of community-dwelling older adults [90,91,92,93,94]. In this study, ADLs were significantly associated with mortality in women and the “self-management” subscale, which was associated in both men and women. The “self-management” subscale contained items that required physical performances. Physical performance and ADLs interacted with each other [95,96]. The association between physical performance and ADLs are shown in Figure 4.

In this study, health status was evaluated via blood tests. Serum albumin, serum creatinine, and serum total cholesterol were associated with mortality in women. Serum creatine is a diagnostic marker of renal function [97,98]. Elevated serum creatine is a risk of mortality for patients with coronary heart disease [99,100,101] and is applicable for community-dwelling older adults [102,103,104]. Elevated total cholesterol is a risk indicator for coronary heart disease. It was also a risk for mortality [105,106,107,108]. Serum total cholesterol reflected dietary habit and nutritional status [108,109,110,111,112,113] yet was not associated with self-assessed chewing ability.

Throughout this study’s results, the effects of health-related factors on mortality were different between men and women. The decline in the number of remaining teeth was a risk factor of mortality only in men [114,115,116]. In women [12,117], it was an insignificant risk [118]. Self-assessed chewing ability was a risk for both men and women [18,19,20,21]; see, only in men [52], and only in women [55]. Serum albumin is a risk factor for mortality both in men and women [52] and only in women [55]. In this study, self-assessed chewing ability was high risk for women and its hazard ratio was 6.7, which was measures with the optimal cut-off point. As shown in Figure 2, a clear survival curve was obtained. All women with declined self-assessed chewing ability died within 3 years of the 10-year follow-up study. Similarly, self-assessed chewing ability was a high risk for men at the age of 80. All men with declined self-assessed chewing ability died within a short period of time. A similar result was presented for women with declined self-assessed chewing ability at the age of 85. The life expectancy of women is longer than men. Therefore, risk factors for mortality depends on the life stage of the subjects. It may be one of the most important aspects for the contractional results between the sexes. Baseline health status, follow-up periods, and statistical models differed between studies. These factors also acted on the contractional results of sex differences.

Self-chewing ability was not associated with serum total cholesterol. Its association with serum albumin in women was very weak. When adjusted with serum albumin, the hazard ratio of self-assessed chewing ability was significant in women, indicating that self-assessed chewing ability independently acted on mortality. Therefore, in women, self-assessed chewing ability and nutritional status were independent. To utilize the oral function, nutritional instructions are indispensable.

The limitation of this study was that it used only a one-point health checkup to predict mortality. More precise risks can be defined by health checkups with regular intervals.

## 5. Conclusions

Self-assessed chewing ability is a strong risk factor for the mortality of women at the age of 90, independent from nutritional status. For older adults, it is useful to address the promotion of healthy food choices together with oral function improvements. However, risk factors for mortality may depend on the life stage of subjects. To investigate risk factors for mortality, the life course approach is necessary.

## Figures and Tables

**Table 1:** Results of Cox’s proportional hazard model analysis for self-assessed chewing ability.

		Women		Men	
		Hazard Ratio (95% CI)	*p*-Value	Hazard Ratio (95% CI)	*p*-Value
Self-assessed Chewing ability	Continuous	1.692 (1.050–2.725)	0.031	0.920 (0.641–1.320)	0.651
	Dichotomous	6.742 (2.09–21.751)	0.001	1.157 (0.469–2.851)	0.751
Number of remaining teeth		1.050 (0.952–1.158)	0.332	1.013 (0.961–1.066)	0.638
Denture use (Use/No use)		2.511 (0.591–10.674)	0.213	1.523 (0.521–4.457)	0.442

**Table 2:** Results of Cox’s proportional hazard model for health status evaluated using blood tests and blood pressure.

		Women		Men	
		Hazard Ratio (95% CI)	*p*-Value	Hazard Ratio (95% CI)	*p*-Value
Hepatic function	AST (U/L)	1.036 (0.985–1.090)	0.173	1.007 (0.984–1.030)	0.571
	ALT (U/L)	1.030 (0.970–1.093)	0.336	0.980 (0.924–1.039)	0.497
	γ-GTP (U/L)	1.023 (0.985–1.061)	0.235	1.005 (0.982–1.028)	0.665
	Total protein (g/dL)	1.252 (0.506–3.106)	0.627	2.725 (0.953–7.752)	0.061
	Albumin (g/dL)	3.333 (1.09–10.101)	0.034	2.070 (0.475–9.009)	0.332
Lipid metabolism	Total cholesterol (mg/dL)	1.073 (1.003–1.032)	0.021	1.007 (0.992–1.022)	0.386
	Try glyceride (mg/dL)	0.999 (0.994–1.004)	0.735	0.999 (0.990–1.007)	0.807
	HDL (mg/dL)	1.026 (0.998–1.054)	0.073	0.991 (0.963–1.020)	0.555
	LDL (mg/dL)	1.013 (0.997–1.029)	0.108	1.014 (0.996–1.032)	0.130
Renal function	Creatinine (mg/dL)	12.770 (1.627–100.211)	0.015	1.372 (0.364–5.165)	0.640
Bone metabolism	Calcium (mg/dL)	1.057 (0.503–2.208)	0.883	2.096 (0.541–8.130)	0.284
	Phosphate (mg/dL)	1.474 (0.695–3.128)	0.312	1.314 (0.620–2.785)	0.475
	Calcium/Phosphate	1.471 (0.657–3.290)	0.348	1.412 (0.723–2.762)	0.312
Carbohydrate metabolism	Glucose (mg/dL)	1.002 (0.995–1.008)	0.654	1.004 (0.997–1.011)	0.276
	HbA1c (%)	1.165 (0.692–1.961)	0.565	1.023 (0.712–1.469)	0.903
Immune function	IgG (mg/dL)	1.000 (0.999–1.001)	0.834	0.999 (0.998–1.000)	0.231
	IgA (mg/dL)	1.001 (0.998–1.003)	0.705	0.996 (0.993–0.999)	0.017
	IgM (mg/dL)	0.993 (0.984–1.002)	0.108	1.003 (0.993–1.012)	0.601
Blood pressure	Systolic pressure (mmHg)	0.990 (0.971–1.010)	0.330	0.993 (0.972–1.014)	0.514
	Diastolic pressure (mmHg)	0.998 (0.974–1.024)	0.901	1.008 (0.977–1.040)	0.611

AST: aspartate transaminase, ALT: alanine aminotransferase, γ-GTP: γ-glutamic pyruvic transaminase, HDL: high-density lipoprotein, LDL: low-density lipoprotein, HbA1c: hemoglobin A1c.

**Table 3:** Results of Cox’s proportional hazard model analysis for the TMIG index.

	Women		Men	
	Hazard Ratio (95% CI)	*p*-Value	Hazard Ratio (95% CI)	*p*-Value
TMIG Index	1.124 (1.020–1.240)	0.019	1.023 (0.921–1.136)	0.670
Self-management (≤4 points/>5 points)	2.119 (1.003– 4.643)	0.048	5.882 (1.822–18.878)	0.003
Intellectual activity (≤2 points/>3 points)	1.484 (0.801–1.249)	0.210	1.304 (0.451–3.774)	0.624
Social role (≤2 points/>3 points)	1.669 (0.900–3.096)	0.104	1.107 (0.351–2.489)	0.806

**Table 4:** Results of Cox’s proportional hazard model for physical performance.

		Women		Men	
		Hazard Ratio (95% CI)	*p*-Value	Hazard Ratio (95% CI)	*p*-Value
		1.072 (1.005–1.143)	0.034	1.043 (0.972–1.119)	0.245
One-legged standing time with eyes open (min)		0.999 (0.916–1.089)	0.977	1.034 (0.945–1.131)	0.461
Stepping	Mean of right and left (steps/10s)	1.019 (0.982–1.057)	0.323	1.027 (0.974–1.083)	0.326
	Maximum of right and left (steps/10s)	1.026 (0.989–1.064)	0.174	1.034 (0.979–1.093)	0.230
	Both legs (steps/10s)	1.009 (0.991–1.028)	0.323	1.013 (0.987–1.041)	0.326
5 m walk	Number of steps	1.012 (0.988–1.037)	0.315	1.035 (0.929–1.153)	0.530
	Time (s)	1.042 (0.992–1.094)	0.101	1.056 (0.917–1.215)	0.451
	Distance (m)	0.815 (0.414–1.606)	0.555	0.945 (0.457–1.952)	0.878

## Supplementary Materials

The following are available online at https://www.mdpi.com/1660-4601/17/24/9584/s1, Figure S1. Diagram of the study design; Figure S2: Survival curves for chewable dried scallops and Konnyaku jelly; Figure S3: Survival curves by subscales of ADLs; Table S1: Descriptive statistics of the variables analyzed in this study; Table S2: Three parameter logistic model for self-assessed chewing ability; Table S3: Hazard ratios of 15 types of food; Table S4: Adjusted hazard ratios of self-assessed chewing ability and ADLs by serum albumin, total cholesterol, and creatine.
