# Supplementary material for: Mortality- and Health-Related Factors in a Community-Dwelling of Oldest-Older Adults at the Age of 90: A 10-Year Follow-Up Study
Source: Int J Environ Res Public Health. 2020 Dec 21;17(24):9584. doi: 10.3390/ijerph17249584 (PMC7768389; doi:10.3390/ijerph17249584)
Supplement: Supplementary file 1 [file ijerph-17-09584-s001.pdf]

Supplemental materials

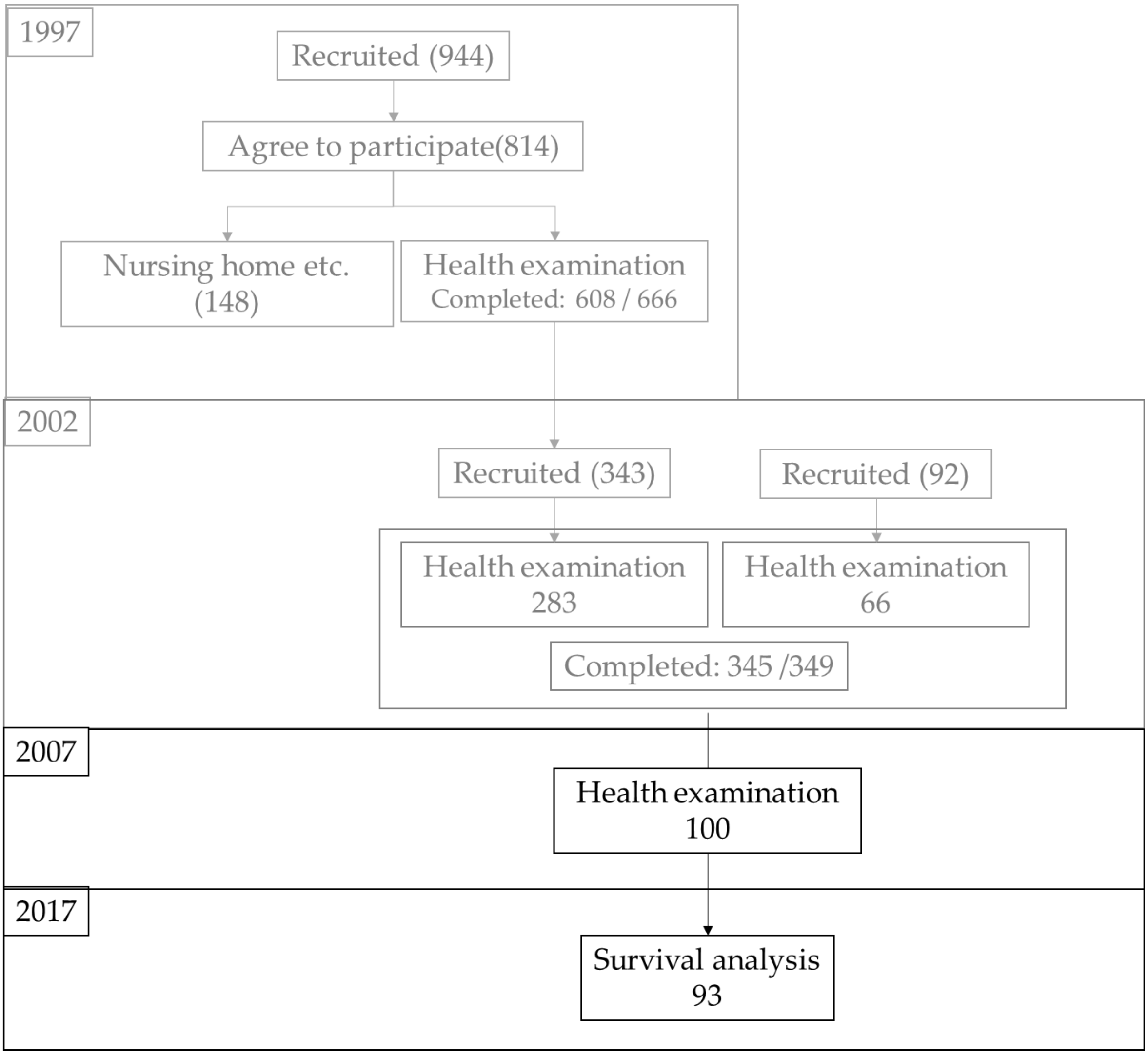

Figure S1. Diagram of the study design
In this study, data of box in black were used for analysis.

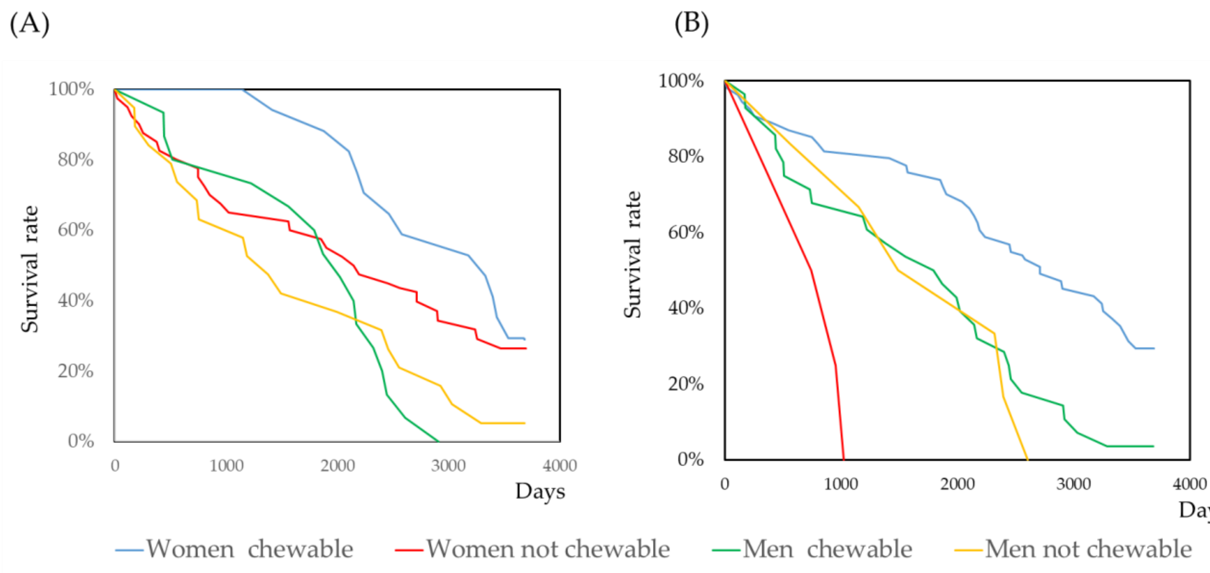

Figure S2. Survival curves by chewable of "Dried scallops" and "Konnyaku-jelly"

(A): Survival curve for Dried scallops (B) Survival curve for Konnyaku-jelly. Subjects participated in this study were classified by chewable or not for "Dried scallops" and "Konnyaku-jelly".
Statistical significance for "Dried scallops" evaluated by Log Rank test, Breslow tests, Tarone-Ware tests were P=0.229, P=0.053, and P=0.106 for woman and P=574, P=0.642, and P=0.973 for men, respectively. Statistical significance for and "Konnyaku-jelly" by Log Rank test, Breslow tests, Tarone-Ware tests were P=<0.001, P=0.001, and P<0.001 for woman and P=0.751, P=0.828, and P=0.992 for men, respectively.

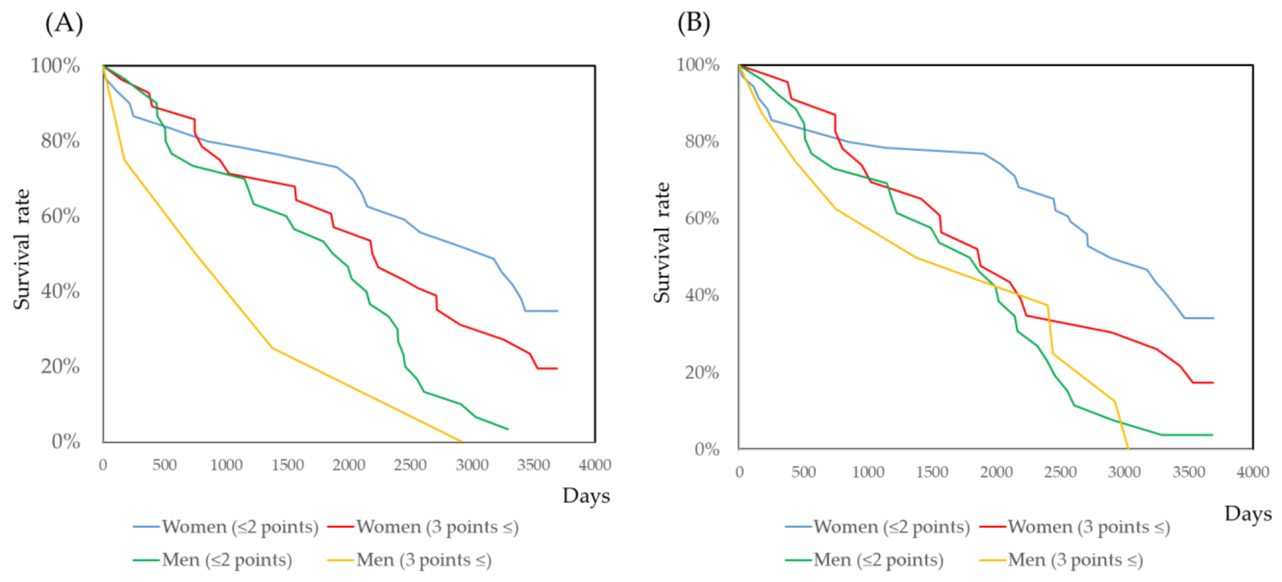

Figure S3. Survival curves by subscales of ADLs

Subjects participated in this study were classified by the "Intellectual activity" and "Social role" of the subscales of TIMG index. Statistical significance for "Intellectual activity" evaluated by Log Rank test, Breslow tests, Tarone-Ware tests were P=0.208, P=0.293, and P=0.241 for woman and P=0.823, P=0.414, and P=0.507 for men, respectively. Statistical significance for "Social role evaluated" by Log Rank test, Breslow tests, Tarone-Ware tests were P=0.100, P=0.120, and P=0.102 for woman and P=0.806, P=0.903, and P=0.800 for men, respectively.

Table S1. Descriptive statistics of the variables analyzed in this study

		Women			Men			Total		
		Mean +/- SD	Median (25th -75th)	Normality	Mean +/- SD	Median (25th -75th)	Normality	Mean +/- SD	Median (25th -75th)	Normality
ADL (TIMG index)		6.4 +/- 3.6	6 (3 - 9)	0.135	4 +/- 3.5	3 (1 - 6)	0.014	5.4 +/- 3.7	5 (2 - 9)	0.001
Self-assessed chewing ability		0.021 +/- 0.783	- 0.054 (- 0.564 - 0.695)	0.004	0.116 +/- 0.862	0.192 (- 0.394 - 0.956)	0.021	0.06 +/- 0.813	0.192 (- 0.413 - 0.714)	<0.001
Health status	BMI	23.1 +/- 3.3	22.4 (20.7 - 26.1)	0.083	22.6 +/- 3.5	23.1 (19.5 - 26)	0.200	22.9 +/- 3.3	22.7 (20.3 - 26)	0.07
	AST (U/L)	24.8 +/- 6.5	24 (21 - 27.8)	0.002	29.3 +/- 17.5	25 (20 - 32)	<0.001	26.7 +/- 12.4	24 (21 - 29)	<0.001
	ALT (U/L)	14.1 +/- 5.0	13 (10.3 - 17)	0.200	17 +/- 6.6	15 (12 - 21)	0.025	15.3 +/- 5.9	14 (11 - 18)	<0.001
	γ- GTP (U/L)	18 +/- 9.1	14 (12 - 20)	<0.001	26.4 +/- 17.2	22 (15 - 32)	0.008	21.5 +/- 13.6	17 (14 - 26)	<0.001
	Total protein (g/dL)	7.2 +/- 0.4	7.2 (7 - 7.4)	0.043	7.2 +/- 0.5	7.2 (6.9 - 7.4)	0.114	7.2 +/- 0.4	7.2 (7 - 7.4)	0.009
	Albumin (g/dL)	4.2 +/- 0.2	4.2 (4.1 - 4.4)	<0.001	4.1 +/- 0.2	4.1 (3.9 - 4.3)	0.178	4.1 +/- 0.2	4.2 (4 - 4.3)	<0.001
	Total cholesterol (mg/dL)	197 +/- 27	198 (180 - 214)	0.200	173 +/- 28	172 (154 - 188)	.200*	187 +/- 30	186 (162 - 208)	0.389
	Try glyceride (mg/dL)	128 +/- 62	114 (71 - 165)	0.006	92 +/- 43	88 (65 - 106)	0.003	113 +/- 57	100 (70 - 131)	<0.001
	HDL (mg/dL)	56 +/- 14	53 (46 - 63)	0.200	51 +/- 15	47 (42 - 56)	0.001	54 +/- 14	50 (43 - 62)	<0.001
	LDL (mg/dL)	116 +/- 23	112 (98 - 135)	0.200	102 +/- 25	97 (85 - 121)	0.200	110 +/- 25	108 (92 - 130)	0.811
	Creatinine (mg/dL)	0.8 +/- 0.2	0.8 (0.6 - 0.9)	0.008	1 +/- 0.3	0.9 (0.8 - 1.1)	0.103	0.8 +/- 0.2	0.8 (0.7 - 0.9)	<0.001
	Calcium (mg/dL)	9.2 +/- 0.5	9.2 (8.9 - 9.4)	0.003	9.1 +/- 0.3	9.1 (8.9 - 9.3)	0.141	9.2 +/- 0.4	9.1 (8.9 - 9.4)	<0.001
	Phosphate (mg/dL)	3.4 +/- 0.5	3.5 (3 - 3.7)	0.200	3 +/- 0.4	3 (2.7 - 3.3)	0.200	3.2 +/- 0.5	3.3 (2. 9- 3.6)	0.838
	Calcium /Phosphate	2.8 +/- 0.4	2.7 (2.5 - 3)	0.200	3.1 +/- 0.5	3 (2.8 - 3.4)	0.200	2.9 +/- 0.5	2.8 (2.6 - 3.2)	0.023
	Glucose (mg/dL)	125 +/- 40	113 (98 - 144)	<0.001	143 +/- 59	127 (99- 149)	<0.001	133 +/- 49	118 (99 - 146)	<0.001
	HbA1c (%)	5.4 +/- 0.6	5.2 (5 - 5.6)	0.001	5.7 +/- 1.1	5.4 (5.1 - 5.9)	0.002	5.5 +/- 0.9	5.2 (5.1 - 5.7)	<0.001

	IgG (mg/dL)	1401 +/- 289	1376 (1209 - 1538)	0.166	1537 +/- 269	1551 (1323 - 1775)	0.200	1458 +/- 287	1429 (1283 - 1626)	0.508
	IgA (mg/dL)	277.1 +/- 101	259 (202.5 - 355.8)	0.077	327.8 +/- 123.4	307 (239- 423)	0.200	298.1 +/- 112.9	272 (204- 383)	0.015
	IgM (mg/dL)	95.9 +/- 32.3	98 (69.3 - 119.5)	0.200	80.9 +/- 37.3	74 (51- 107)	0.200	89.7 +/- 35	94 (60- 114)	0.096
	Systolic pressure (mmHg)	148.3 +/- 20.7	147 (135.3 - 155.8)	0.015	144.2 +/- 18.4	141 (133- 152)	0.145	146.6 +/- 19.7	145 (133- 155)	<0.001
	Diastolic pressure (mmHg)	71.2 +/- 13.3	70 (62.3 - 82.5)	0.200	72 +/- 11.2	69 (64- 81)	0.064	71.5 +/- 12.4	70 (63- 81)	0.419
Physical performance	Hand grip strength (Kg)	16.5 +/- 4.1	17.5 (14.1 - 19)	0.008	25 +/- 5.7	25 (20.5- 29)	0.200	20 +/- 6.4	19 (16.5- 23.5)	0.133
	One-leg standing time with eyes open (min)	3.5 +/- 4.3	2.3 (1.3 - 3.9)	<0.001	4.6 +/- 3.8	3.1 (2.5- 6)	<0.001	3.9 +/- 4.1	2.6 (1.6- 4.5)	<0.001
	Stepping Mean of right and left (Kg)	26.5 +/- 8.6	26.3 (19.3 - 33.4)	0.200	33.6 +/- 7.7	34.5 (26.5- 39.5)	0.200	29.4 +/- 8.9	29.5 (22- 36.5)	0.33
	Maximum of right and left (Kg)	27.6 +/- 9.4	27 (19.5 - 34.8)	0.200	34.4 +/- 7.4	35 (28- 40)	.200*	30.4 +/- 9.2	30 (24- 37)	0.518
	Both legs (Kg)	53 +/- 17.3	52.5 (38.5 - 66.8)	0.200	67.2 +/- 15.3	69 (53- 79)	0.200	58.9 +/- 17.8	59 (44- 73)	0.33
	5m walk Number of steps	16.1 +/- 6.9	15.5 (11 - 19)	0.022	11.9 +/- 3.3	11 (10- 13)	0.043	14.4 +/- 6.1	13 (11- 16)	<0.001
	Time (sec)	8 +/- 4.9	6.1 (4.8 - 9.3)	<0.001	5.6 +/- 1.9	5.1 (4.3- 6.8)	0.068	7 +/- 4.1	5.8 (4.4- 8.6)	<0.001
	Distance (m)	5.5 +/- 0.5	5.3 (5.1 - 5.9)	<0.001	5.4 +/- 0.5	5.2 (5- 5.9)	<0.001	5.5 +/- 0.5	5.2 (5.1- 5.9)	<0.001

Normality was calculated by Kolmogorov–Smirnov test. P>0.05 indicate the value was normally distributed. For physical fitness, tests were dangerous or burden for the some subjects. Number of subjects escaped the tests were as follows: had grip(1), stepping(3), and 5m walk(8). AST: Aspartate transaminase, ALT: Alanine

aminotransferase, γ-GTP: γ-Glutamic Pyruvic Transaminase, HDL: High density lipoprotein, LDL: Low density lipoprotein. HbA1c: Hemoglobin A1c.

Table S2. Three parameter logistic model for self-assessed chewing ability

	Discrimination	Difficulty	Guessing
Peanuts	2.477	-0.093	0.219
Yellow pickled radish	78.887	-0.334	0.413
Hard rice crackers	1.690	-0.896	<0.001
French bread	2.615	0.052	<0.001
Beefsteak	2.942	0.214	0.094
Octopus in vinegar	4.421	0.060	0.148
Pickled shallots	4.125	-0.772	0.000
Dried scallops	21.279	0.414	0.184
Dried cuttlefish	34.487	0.358	0.102
Squid-sashimi	3.817	-0.304	0.328
Konnyaku-jelly	44.482	-1.087	<0.001
Tubular roll of boiled fish paste	3.489	-1.831	<0.001
Steamed rice	40.422	-2.098	<0.001
Tuna sashimi	2.114	-1.577	<0.001
Grilled eel	2.178	-1.114	0.149

"Discrimination" indicates the steepness of the item response curve, "Difficulty" indicate the location of item response curve and "Guessing" indicates the baseline of vertical values of item response curve.

Table S3. Hazard ratios of 15 type foods

	Women		Men	
	Hazard ratio (95% CI)	P-value	Hazard ratio (95% CI)	P-value
Peanuts	1.672 (0.887 - 3.145)	0.112	1.527 (0.746 - 3.125)	0.247
Yellow pickled radish	1.883 (0.979 - 3.623)	0.058	1.515 (0.684 - 3.356)	0.307
Hard rice crackers	1.109 (0.551 - 2.232)	0.772	1.546 (0.726 - 3.289)	0.258
French bread	1.524 (0.796 - 2.915)	0.204	1.572 (0.764 - 3.236)	0.219
Beefsteak	1.466 (0.761 - 2.817)	0.253	1.808 (0.892 -3.676)	0.100
Octopus in vinegar	1.307 (0.695 - 2.457)	0.406	1.222 (0.610 - 2.451)	0.571
Pickled shallots	1.645 (0.853 - 3.165)	0.137	1.192 (0.527 - 2.695)	0.673
Dried scallops	2.105(1.025 - 4.329)	0.043	1.171 (0.587 - 2.331)	0.655
Dried cuttlefish	1.435 (0.726 - 2.833)	0.298	0.812 (0.392 - 1.684)	0.575
Squid-sashimi	1.059 (0.555 - 2.020)	0.862	2.358 (0.872 - 6.369)	0.091
Konnyaku-jelly	7.092 (2.151 - 23.256)	0.001	1.157 (0.469 - 2.849)	0.751
Tubular roll of boiled fish paste	2.710 (0.817 - 9.009)	0.103	0.839 (0.253 - 2.778)	0.774
Steamed rice	4.484 (0.578 - 34.483)	0.151	0.656 (0.155 - 2.770)	0.566
Tuna sashimi	1.188 (0.523 - 2.695)	0.682	0.839 (0.253 - 2.778)	0.774
Grilled eel	1.647 (0.778 - 3.484)	0.193	0.926 (0.352 - 2.433)	0.876

Table S4. Adjusted hazard ratios of self-assessed chewing ability and ADLs by serum albumin, total cholesterol and creatine.

	Women		Men	
	Hazard ratio (95% CI)	P-value	Hazard ratio (95% CI)	P-value
Albumin				
Albumin(g/dL)	4.785 (1.418 - 16.129)	0.012	2.203 (0.509 - 9.524)	0.291
Chewing ability	1.812 (1.122 - 2.924)	0.015	1.104 (0.753 - 1.618)	0.613
Albumin(g/dL)	3.003 (0.951 - 9.434)	0.061	1.957 (0.433 -8.850)	0.383
ADLs	0.905 (0.822 - 0.997)	0.042	0.980 (0.880 -1.093)	0.723
Total Cholesterol				
Total Cholesterol(mg/dL)	1.016 (1.001 - 1.031)	0.035	1.007 (0.992 - 1.021)	0.376
Chewing ability	1.538 (0.954 - 2.481)	0.077	1.071 (0.736 - 1.558)	0.721
Total Cholesterol(mg/dL)	1.016 (1.003 - 1.031)	0.018	1.006 (0.992 - 1.021)	0.391
ADLs	0.894 (0.812 - 0.983)	0.022	0.971 (0.873 - 1.080)	0.589
Creatine				
Creatine(mg/dL)	8.616 (1.045 - 71.035)	0.045	1.329 (5.183 - 0.341)	0.682
Chewing ability	1.473 (0.883 - 2.457)	0.137	1.041 (0.715 - 1.515)	0.834
Creatine(mg/dL)	9.079 (1.155 - 71.361)	0.036	1.602 (0.404 - 6.352)	0.502
ADLs	0.907 (0.820 - 1.004)	0.060	0.959 (0.855 - 1.075)	0.468
